# Aspirin/PLGA coated 3D-printed Ti-6Al-4V alloy modulate macrophage polarization to enhance osteoblast differentiation and osseointegration

**DOI:** 10.1007/s10856-022-06697-w

**Published:** 2022-10-08

**Authors:** Yapeng You, Wanmeng Wang, Ying Li, Yunjia Song, Jian Jiao, Yao Wang, Bo Chen, Jialin Liu, Hui Qi, Yu Liang

**Affiliations:** 1 School of Stomatology, Hospital of Stomatology, Tianjin Medical University, Tianjin, 300070 China; 2 The Affiliated Stomatology Hospital of Kunming Medical University, Kunming, Yunnan 650100 China

## Abstract

Although titanium (Ti) and Ti-based alloy have been widely used as dental and orthopedic implant materials, its bioinertness hindered the rapid osseointegration. Therefore, it is recommended to acquire ideal topographic and chemical characteristics through surface modification methods. 3D printing is a delicate manufacture technique which possesses superior controllability and reproducibility. While aspirin serve as a well-established non-steroidal anti-inflammatory agent. Recently, the importance of immune system in regulating bone dynamics has attracted increasing attention. We herein superimposed the aspirin/poly (lactic–co–glycolic acid) (ASP/PLGA) coating on the 3D-printed Ti-6Al-4V surface with uniform micro-structure to establish the Ti64-M-ASP/PLGA substrate. Scanning electron microscopy (SEM), x-ray photoelectron spectroscopy (XPS) and contact angle test confirmed the successful fabrication of the Ti64-M-ASP/PLGA substrate, with increased wettability and sustained release pattern of ASP. Compared with the Ti64 base material, the Ti64-M-ASP/PLGA substrate showed enhanced M2 and depressed M1 genes and proteins expressions in macrophages. The novel Ti64-M-ASP/PLGA substrate also displayed enhanced osteoblast proliferation, adhesion, extracellular mineralization ability and osteogenic gene expressions when cultured with macrophage conditioned medium in vitro. Furthermore, rat femora implantation model was used for in vivo evaluation. After 4 weeks of implantation, push out test, micro-computed tomography (micro-CT) and histological analyses all confirmed the superior osseointegration capabilities of the Ti64-M-ASP/PLGA implant than the other groups. Our study revealed the synergistic role played by 3D-printed micro topography and immunoregulatory drug aspirin in promoting osteogenesis in vitro and accelerating osseointegration in vivo, thus providing a promising method for better modifying the implant surface.

**Figure:**
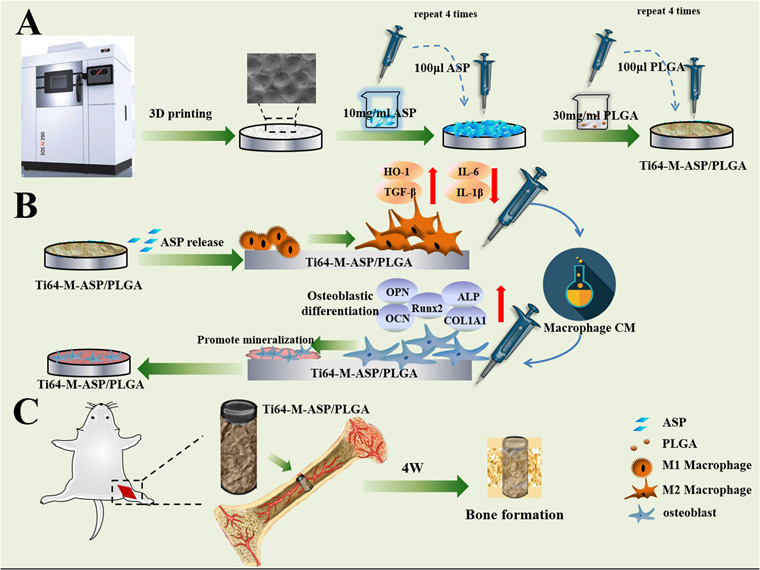
Graphical abstract

Graphical abstract

## Introduction

Achieving ideal osseointegration is crucial for implant success in both dental and orthopedic fields. Ti and Ti-based alloy have been extensively used as implant materials for decades because of their excellent biocompatibility, appropriate mechanical properties and corrosion resistance [1]. However, their inherent bioinertness impaired the rapid initial cell adhesion and subsequent osseointegration [2]. Since surface micro-structure of implant material plays a crucial role in achieving satisfied biocompatibility, numerous surface modification methods have been employed to improve the bioactivity of Ti alloys including sandblast [3], plasma spraying [4], sol-gel method [5], etc. It has been reported that bone cells are prone to adhere to surface with 200–500 μm pore size [6]. In addition, it is suggested that pore size around 300 μm can promote the formation of new bone and capillaries [7]. Therefore, it is promising to fabricate 300 μm sized concave micro-structure on the Ti alloy to promote osteoblast cell adhesion and subsequent osteogenic ability.

The main problem for fabricating such micro-architecture lies in that traditional manufacture methods, such as power sintering, foam fabrication and plasma spray coating, are unable to produce highly controlled, reproducible surface topography as designed [8]. Fortunately, with the advent of 3D-printing technique, this problem can be solved and the structure design can be precisely controlled by adjusting computer-assisted design and computer-assisted manufacturing (CAD/CAM) parameters [9]. Herein, we prepared the uniform 300 μm concave micro-structure on the Ti alloy surface using selective laser melting (SLM) method.

Although many research concerning 3D-printed Ti and Ti-alloy implants have been performed, most of them focused on the mechanical aspect instead of surface micro-structure of the implants [10–12]. However, after implant is inserted into the bone, cells firstly contact with the implant surface, which will determine the cell response such as cell adhesion, proliferation, differentiation and osseointegration [13]. Therefore, in this work, we fabricated the 3D-printed Ti alloy surface with evenly arranged 300 μm sized concave micro-structure and explore the influence of the modified surface on cells.

When evaluating the cell reactions of biomaterials, plenty of studies focused on osteoblast cells [14] and mesenchymal stem cells [15]. However, the discrepancy between in vitro and in vivo experiment for implant materials is frequently observed [16]. The reason lies in that the in vivo environment has not been fully appreciated and some potential crucial elements, such as immunomodulatory aspect, are ignored [17, 18]. Recently, much attention has been paid to the importance of immune response during osteogenesis at the implant-tissue interface [19]. Macrophages, one of the main cell types of immune cells [20], contact with implant surface firstly after implant insertion [21]. Therefore, we herein tend to reveal the important roles macrophages play during osseointegration process.

Moreover, aspirin (ASP) is an inexpensive, chemically stable non-steroidal anti-inflammatory drug (NSAID) [22, 23]. Besides its long-term application as a painkiller after operation [24], ASP also plays an important role in anti-inflammatory effect [25] and osteogenic differentiation [26]. Since oral administration of ASP will reduce the drug concentration reaching the local inflammatory area [22], local application is recommended. In the case of local administration, on one hand, the short half-life of ASP requires repeated administration for maintaining long-term efficacy [27]. On the other hand, ASP overdoses can give rise to adverse drug reactions including ulcers and gastric damage [28]. We previously reported that the icariin/aspirin composite coating on the TiO_2_ nanotube surface could induce immunomodulatory effect of macrophage and improve osteoblast activity [29]. However, the release period of aspirin only lasted 7 days. Therefore, it is necessary to construct a local drug delivery system to achieve sustained release of ASP to exert its long-term function and avoid adverse effects at the same time.

Poly lactic-co-glycolic acid (PLGA), which serve as a green, environmentally friendly and degradable material [30], is widely used as a drug-loading material [31]. PLGA has favorable mechanical properties [32] and drug-carrying capacity [33] which can be used as a non-toxic coating material on metal surface [34] and achieve effective drug dose during local administration [35]. Thus, we employed PLGA to acquire controlled release of ASP.

In the present study, we deposited the ASP/PLGA coating on the 3D-printed Ti-6Al-4V alloy with uniform concave micro-structured surface to fabricate the Ti64-M-ASP/PLGA substrate. The release profile of the established ASP coating was evaluated. Afterwards, the cell proliferation, cytotoxicity, cell morphology and pro-inflammatory (M1) and pro-regenerative (M2) marker gene and protein expressions of macrophages were evaluated. In addition, osteoblast cell responses including cell proliferation, cytotoxicity, cell morphology, osteogenesis-related gene level and cell calcification were analyzed when cultured with macrophage conditioned medium (CM). Furthermore, animal experiment was utilized to confirm the in vivo osseointegration effects.

Our hypothesis is that the ASP/PLGA coating can induce macrophage M2 polarization and construct ideal immune microenvironment to promote bone regeneration. The established Ti64-M-ASP/PLGA substrate could combine the ASP/PLGA coating with the preexisted micro-structured surface to acquire the functionalized composite surface and generate synergistic effects to promote osteoblast osteogenesis and facilitate osseointegration.

## Material and methods

### Specimen fabrication

#### Fabrication of microstructure using 3D-printing technique

**Fig. 1:**
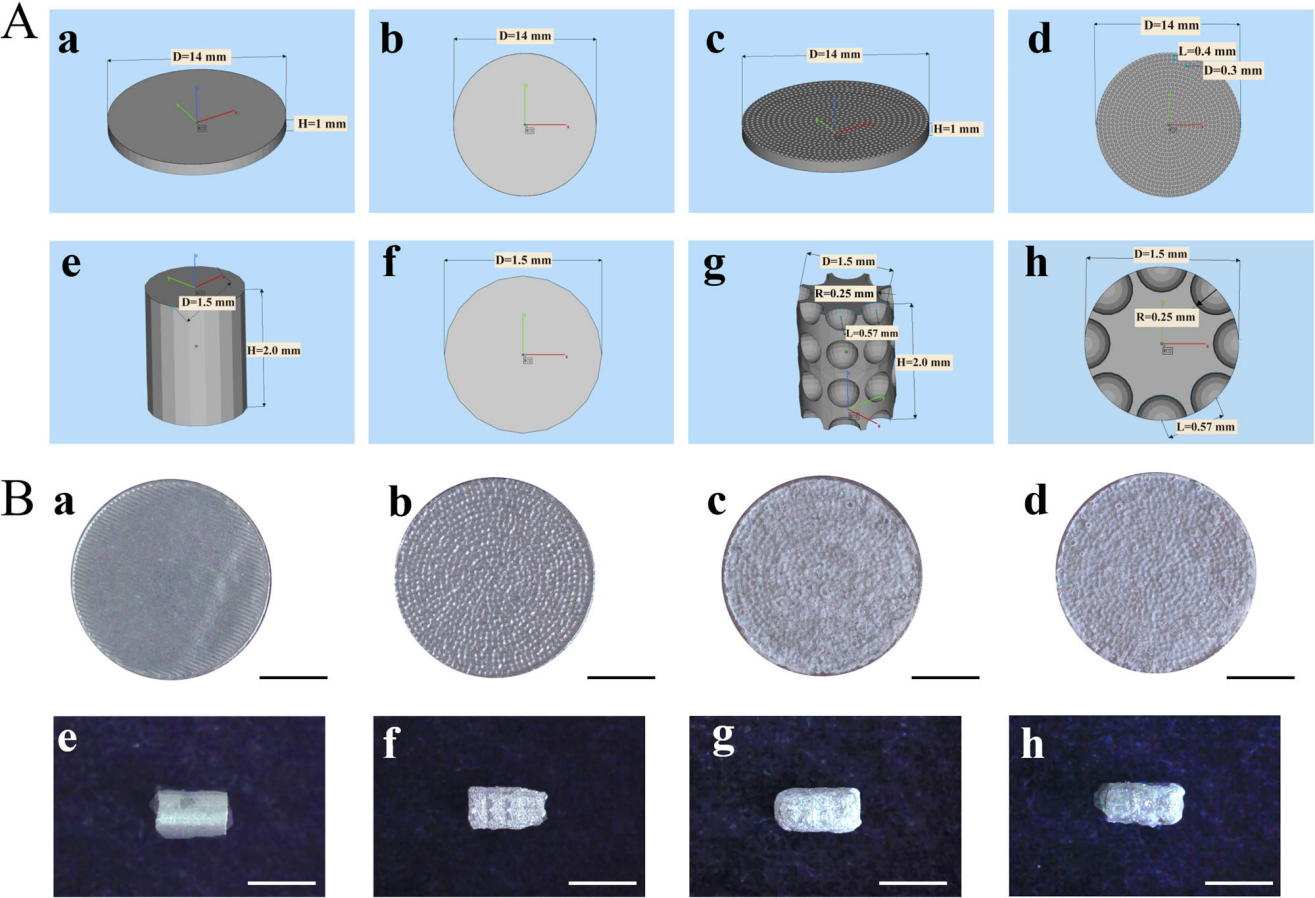
Designed 3D models and fabricated entities of different samples. **A** 3D models of Ti64 and Ti64-M. Figure b, d, f, h are the top view of figure a, c, e, g, respectively. **B** Macro surface morphology of different samples. Scale bars indicate 5 mm (a–d) and 2 mm (e–h)

The 3D models designed using Magics software (ver. 19.0, Materialise company, Leuven, Belgium) were displayed in Fig. 1A. Disc form models (Fig. 1A, a–d) (diameter, 14 mm; thickness, 1.5 mm) were designed for in vitro test, while cylinder form models (Fig. 1A, e–h) (diameter 1.5 mm; height, 2 mm) were designed for in vivo experiment. Both 3D discs and cylinder-shaped models without special surface structure were named as Ti64 (Fig. 1A-a, b, e, f). While the models with evenly distributed 300 μm-diameter concave micro-structures were annotated as Ti64-M (Fig. 1A-c, d, g, h). All designed models were saved in STL format and fabricated by selective laser melting system (SLM, EOS M290, Munich, Germany), with printing power of 200 W, laser scanning speed of 950 mm/s and laser spot diameter of 100 μm under inert argon atmosphere. All samples were ultrasonically cleaned in acetone, ethyl alcohol and deionized water for 10 min successively and dried in air.

#### Construct ASP coating on the micro-structured Ti alloy surface

ASP and PLGA was provided by Sigma-Aldrich (St. Louis, MO, USA). The 10 mg/ml ASP and 30 mg/ml PLGA solution were prepared by resolving the solute in acetone, respectively and mix to acquire a homogeneous solution. PLGA and ASP/PLGA coating were superimposed onto the Ti64-M surface using drop-coating method. Briefly, 100 μl PLGA solution were spread on the Ti64-M surface evenly, and repeated 4 times to construct the Ti64-M-PLGA surface. The Ti64-M-ASP/PLGA substrate was fabricated by applying 100 μl ASP solution on the Ti64-M surface evenly and repeated 4 times firstly. Then, 100 μl PLGA solution were superimposed to the ASP coated surface, repeated 4 times to obtain the Ti64-M-ASP/PLGA substrate.

### Surface characterization and drug release test

The macro surface morphology of various samples was observed using stereomicroscope (S9i; Leica Microsystem, Tokyo, Japan), while scanning electron microscopy (SEM; SU8010, Hitachi, Tokyo, Japan) was used to observe the micro surface topography of different samples. X-ray photoelectron spectroscopy (XPS; AXIS Nova; Kratos Analytical, Manchester, UK) was used to detect the elemental composition and valence states of various surfaces. The hydrophilicity was tested by dynamic contact angle tester (JAW-360A, Chongda intelligent technology, Xiamen, China). All samples were measured for three times.

The high-performance liquid chromatography system (HPLC; 1100 Series; Agilent Technologies, Santa Clara, CA, USA) were used for detecting drug release profiles of ASP. Supernatant from each group were collected every day and continued for 30 days. The concentration of ASP was analyzed according to the calibration curve.

### Behaviors of macrophages cultured on different samples

#### Cell culture

RAW 264.7 macrophage cells (American Type Culture Collection, ATCC, Manassas, VA, USA) were cultured in Dulbecco’s Modified Eagle’s Medium (DMEM; Gibco, Carlsbad, CA, USA) supplemented with 10% fetal bovine serum (FBS; Gibco, Carlsbad, CA, USA) and 100 μg/ml penicillin and 100 μg/ml streptomycin (Gibco, Carlsbad, CA, USA) in an incubator (Thermo Fisher Scientific, MA, USA) with 5% CO_2_ at 37 °C. All samples used for cell culture were sterilized by irradiation with 25 kGy Cobalt 60 for 30 min, then were placed in 24-well plate. Cells were seeded at a density of 1 × 10^4^ per well. Lipopolysaccharides (LPS, 1 µg/mL; Sigma-Aldrich, MO, USA) were added to each well after 24 h, this point was defined as day 0.

#### Cell proliferation

The cell proliferation ability was tested by a cell counting kit-8 assay (CCK-8, New Cell and Molecular Biotech, Suzhou, Zhejiang, China) on day 1, 3, 5, 7, following the manufacturer’s instructions.

#### Cytotoxicity

On 1, 3, 5 and 7 days, the cytotoxicity of different samples was measured using the Lactate dehydrogenase kit (LDH kit; Solarbio, Beijing, China), in accordance with the manufacturer’s instruction.

#### Cell Morphology

After 24 h of incubation, samples were fixed with 2.5% glutaraldehyde solution (Solarbio, Beijing, China) at 4 °C overnight, and 1% osmic acid 1 h, respectively. Then samples were dehydrated by sequential ethanol solutions, dried by critical point dryer (EM CPD030, Leica Microsystems, Wetzlar, Germany). After gold sputtering, cell morphologies were observed and photographed by SEM (SU8010, Hitachi, Tokyo, Japan).

#### Pro-inflammatory (M1) and pro-regenerative (M2) marker gene expression

On day 3, Total RNA from cells on various surfaces was extracted by TRIzol (Thermo Fisher Scientific, Waltham, MA, USA). Nanodrop spectrophotometer (NanoDrop Technologies, Wilmington, DE, USA) was used to detect the RNA concentration and purity at 260 nm. Mouse mRNA encoding genes for *interleukin-6* (*IL-6*), *interleukin-1 beta* (*IL–1β*), *transforming growth factor-beta* (*TGF-β*) and *heme oxygenase-1* (*HO–1*) were selected, *glyceraldehyde–3–phosphate dehydrogenase* (*GAPDH*) was used as an internal control. Reverse transcription and quantitative real-time polymerase chain reaction (qPCR) was performed and data were calculated by the 2^−ΔΔCt^ method. Primers for interested genes and the housekeeping gene were shown in Table 1.

**Table 1:** Primers for pro-inflammatory (M1) and pro-regenerative (M2) marker genes

Gene	Gene bank ID	DNA primer	sequence	Size (bp)
*IL-6*	NM_031168.2	Forward	5′-CCAGAGATACAAAGAAATGATGG-3′	88
		Reverse	5′-ACTCCAGAAGACCAGAGGAAAT-3′	
*IL-1β*	NM_008361.4	Forward	5′-TGTGCAAGTGTCTGAAGCAGC-3′	129
		Reverse	5′-TGGAAGCAGCCCTTCATCTT-3′	
*TGF-β*	NM_011577.2	Forward	5′-TTGCTTCAGCTCCACAGAGA-3′	183
		Reverse	5′-TGGTTGTAGAGGGCAAGGAC-3′	
*HO-1*	NM_010442.2	Forward	5′-GCCGAGAATGCTGAGTTCATG-3′	86
		Reverse	5′-TGGTACAAGGAAGCCATCACC-3′	
*GAPDH*	NM_008084.3	Forward	5′-GGTGAAGGTCGGTGTGAACG-3′	233
		Reverse	5′-CTCGCTCCTGGAAGATGGTG-3′	

#### Enzyme-linked immunosorbent (ELISA) assay

The pro-inflammatory (M1) and pro-regenerative (M2) marker protein expression levels in cell supernatant were further measured by enzyme-linked immunosorbent (ELISA) kits (ImmunoWay, TX, USA). The concentrations of proteins including interleukin-6 (IL-6), interleukin-1beta (IL–1β), transforming growth factor-beta (TGF-β) and heme oxygenase-1 (HO–1) were determined by OD values and calculated by standard curve.

### Behaviors of MC3T3-E1 cells on various surfaces cultured with macrophage CM

#### Preparation of macrophage CM and osteoblast cells culture

Macrophages were seeded on various surface and stimulated with LPS (1 µg/mL) for the initial three days. The macrophage culture medium was daily collected and 1:1 mixed with osteoblast medium (alpha MEM with 10% FBS and 1% penicillin- streptomycin) to obtain macrophage CM. MC3T3-E1 preosteoblast cells (ATCC, Manassas, VA, USA) were cultured in CM with 5% CO_2_ at 37 °C.

#### Cell proliferation

The CCK-8 kit was used for cell proliferation ability evaluation on day 1, 3, 5 and 7.

#### Cytotoxicity assay

The LDH kit was employed to examine cytotoxicity on 1, 3, 5 and 7 days.

#### Cell morphology

MC3T3-E1 cells were seeded on different samples at a density of 1 × 10^4^ cells per well in 24-well plate and observed on 24 h by SEM.

#### Osteogenesis-related gene levels

MC3T3-E1 cells were cultured for 14 days on different samples and extracted by TRIzol to detect the osteogenesis-related gene expression levels. Mouse mRNA encoding genes for *runt-related transcription factor-2* (*Runx-2*), *alkaline phosphatase* (*ALP*), *collagen type 1 alpha 1* (*COL1A1*), *osteopontin* (*OPN*) and *osteocalcin* (*OCN*) were selected, *GAPDH* was used as an internal control. Reverse transcription and qPCR were performed, and results were calculated by the 2^−ΔΔCt^ method. Target genes and the housekeeping gene primers were shown in Table 2.

**Table 2:** Primers for osteogenesis-related genes

Gene	Gene bank ID	DNA primer	Sequence	Size (bp)
*Runx-2*	NM_009820.5	Forward	5′-CAAGAAGGCTCTGGCGTTTA-3′	82
		Reverse	5′-TGCAGCCTTAAATGACTCGG-3′	
*ALP*	NM_007431.3	Forward	5′-ATCTTTGGTCTGGCTCCCATG-3′	106
		Reverse	5′-TTTCCCGTTCACCGTCCAC-3′	
*COL1A1*	NM_007742.4	Forward	5′-TAAGGGTCCCCAATGGTGAGA-3′	203
		Reverse	5′-GGGTCCCTCGACTCCTACAT-3′	
*OPN*	NM_009263.3	Forward	5′-CTCACATGAAGAGCGGTGAG-3′	174
		Reverse	5′-TCTCCTGGCTCTCTTTGGAA-3′	
*OCN*	NM_007541.3	Forward	5′-GGACCATCTTTCTGCTCACTCTG-3′	131
		Reverse	5′-GTTCACTACCTTATTGCCCTCCTG-3′	
*GAPDH*	NM_008084.3	Forward	5′-GGTGAAGGTCGGTGTGAACG-3′	233
		Reverse	5′-CTCGCTCCTGGAAGATGGTG-3′	

#### Alizarin Red S (ARS**)** Staining and quantification

MC3T3-E1 cells were cultured for 21 days on different samples. After that, samples were rinsed twice with PBS and stained with 2% Alizarin red S (Sigma-Aldrich St. Louis, MO, USA) for 30 min at 37 °C. A stereomicroscope (S9i; Leica Microsystem, Tokyo, Japan) was used to take picture of each group. 10% cetylpyridinium chloride (CPC; Sigma-Aldrich) was used for quantification analysis. Each sample was incubated with 1 mL of CPC for 30 min. After that, 100 μL aliquots containing the extracted dye were shifted to a new 96-well plate to read the absorbance by microplate reader at 562 nm.

### In vivo experiments

#### Surgical procedures

The experiment in this study were approved by the Animal Ethics Welfare Committee (AEWC) of Tianjin Hospital of Itcwm Nankai Hospital (approval no. NKYY-DWLL-2020-147). Thirty-six male Sprague Dawley (SD) rats were randomly divided into four groups (Ti64, Ti64-M, Ti64-M-PLGA and Ti64-M-ASP/PLGA). Each group performed three kinds of experiments including push-out test, micro-CT and histological analyses (*n* = 3 for each experiment). The animals were housed in an environment of 25 °C and 55% humidity in a 12 h alternating light-dark cycle. Surgical procedures were carried out under standard anesthetic and analgesic protocols by intraperitoneal administration of sodium pentobarbital (50 mg/kg body weight). The right hind limbs of rats were shaved and the mid-diaphysis of the femora was exposed. Then, a hole was carefully made perpendicular to the surface of the femur using a motorized dental drill (Φ = 1.5 mm). After that, the implant was inserted into the femur, and the skin and muscle tissues were cleaned and sutured subsequently. Rats were sacrificed 4 weeks after implantation and the bones tissue containing implants were harvested and stored at 10% buffered formalin at 4 °C.

#### Push-out test

To evaluate the strength of bone-implant integration, push-out test was carried out. Briefly, three randomly selected rats in each group were sacrificed after 4 weeks of operation. A mechanical testing machine (Bose ElectroForce 3230; Bose Corporation, USA) with a push rod of 1 mm in diameter was used. The displacement of the implant towards the surrounding bone was at the speed of 1 mm/min until the implant was completely pushed out. Then the load displacement curve was recorded, and the peak value was regarded as the bonding strength value.

#### Micro-computed tomography (micro-CT) analysis

The rat femora including embedded implants from four groups were harvested and scanned by vivaCT 80 micro-CT scanner (Scanco Medical, Brüttisellen, Switzerland). The samples were scanned at 90 kV with the resolution of 10 μm and an exposure time of 200 ms. The 3D images were reconstructed using Nrecon software (ver. 1.6, Skyscan company, Kontich, Belgium). The cylinder-shaped area 1 mm in diameter around the implant is defined as the volume of interest (VOI). Then, quantitative analyses including BV/TV (bone volume/total volume), Tb.Th (trabecular thickness), Tb.N, (trabecular number) and Tb.Sp (trabecular separation) were performed using the CTAn program from the Skyscan company (ver. 1.17, Kontich, Belgium). Each group contains three samples.

#### Histological analysis

Three samples from each group were collected for histological analysis. The rat femora samples with different kinds of implants were immersed in 17% EDTA solution for decalcification and then gradient dehydration by ethanol. Then, samples were embedded into paraffin to cut into 4 μm slices followed by hematoxylin and eosin (H&E) and Masson staining. The samples were observed and photographed with microscope (Olympus IX73, Tokyo, Japan).

### Statistical analysis

The experiments were repeated three times, and the data were expressed as mean ± standard deviation (SD). The one-way ANOVA followed by the Tukey post-hoc test was employed to determine the statistical difference between groups. Values of *p* < 0.05 was regarded as statistical significance. The SPSS software, version 22.0 (SPSS, Chicago, IL, USA), was used for statistics analyses.

## Results and discussion

### Surface characterization and drug release profile

**Fig. 2:**
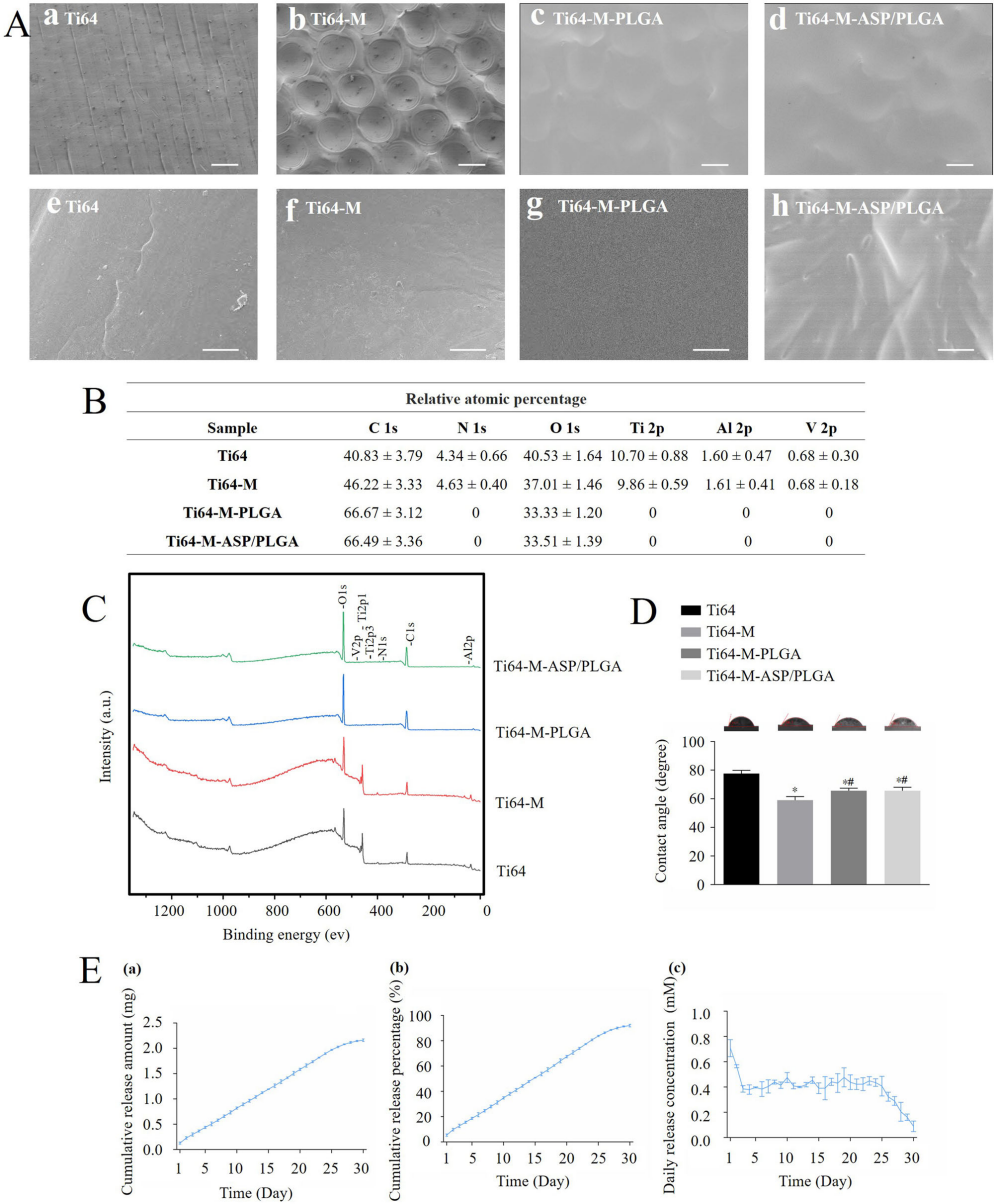
Surface morphology, chemical composition, hydrophilicity and ASP release profile observation. **A** SEM images of different samples. Figure e–h are the magnified SEM pictures of a–d, respectively. Scale bars indicate 300 µm (a–d) and 10 µm (e–h), respectively. **B**, **C** XPS results of different samples. **D** Contact angle measurement of different samples. **E** ASP release profiles of the Ti64-M-ASP/PLGA substrate detected by HPLC. Data are shown as the mean ± standard deviation (*n* = 3). ∗ and # indicate statistical significance *p* < 0.05 *vs* Ti64 and Ti64-M, respectively

Figure 1B showed the surface topography of different surfaces by stereomicroscope. Disc forms (Fig. 1B, a–d) of different samples were used for in vitro test, while cylindrical implants (Fig. 1B, e–h) were prepared for in vivo experiment. From the macro images, the surface of Ti64 is flat and intact, and a concave structure can be observed evenly distributed on the Ti64-M surface. While both the Ti64-M-PLGA and Ti64-M-ASP/PLGA surfaces were covered by a pale white film after PLGA and ASP/PLGA coating application. The surface micromorphology images were obtained with SEM (Fig. 2A). From the SEM images, the Ti64 surface displayed some parallel stripes (Fig. 2A-a), and the Ti64-M surface displayed evenly distributed uniform concave microstructure, with a diameter of about 300 μm produced on the surface, as designed (Fig. 2A-b). While, on both the Ti64-M-PLGA and Ti64-M-ASP/PLGA surfaces, the original concave microstructure was overlaid by membrane of PLGA and ASP/PLGA, respectively, with only slight contour visible (Fig. 2A-c, d). When observed under higher magnification, needle-like ASP crystal structure was observed under the ASP/PLGA film for the Ti64-M-ASP/PLGA substrate (Fig. 2A-h), compared to the smooth and uniform PLGA film on the Ti64-M-PLGA surface (Fig. 2A-g). These results demonstrated the successful preparation of the designed surfaces.

As shown in XPS results (Fig. 2B, C), the spectra of the Ti64 and Ti64-M surfaces include C, O, N, Ti, Al and V, while Ti, Al and V peaks disappeared on the Ti64-M-PLGA and Ti64-M-ASP/PLGA surfaces. This indicates that the PLGA coating has completely overspread the original Ti64 alloy-based material. Moreover, the existence of O element on the Ti64 and Ti64-M surfaces is due to the oxidation reaction on the metal surface, whereas the distinctive peaks of O1s and C1s observed on the Ti64-M-PLGA and Ti64-M-ASP/PLGA surfaces were attributed to the deposition of PLGA coating. In addition, since ASP (C_9_H_8_O_4_) have relatively higher C and lower O element content than PLGA ([C_5_H_8_O_5_]_n_), the similarity XPS results between the Ti64-M-PLGA and Ti64-M-ASP/PLGA substrates may be explained by the total coverage of ASP in the PLGA coating.

In addition to surface chemical composition and topography, hydrophilicity also serve as a key parameter of biomaterials in cell responses, including cell adhesion, proliferation, and differentiation [36, 37]. Figure 2D displayed that the Ti64-M surface revealed the lowest contact angle, which implied that the micro-structure significantly enhances surface hydrophilicity. While contact angles also decreased on both the Ti64-M-PLGA and Ti64-M-ASP/PLGA surfaces, compared to the Ti64 surface. Previous studies proved that wettability of implant surfaces not only activate macrophages [38, 39] but also improve osteoblasts osteogenesis [40]. Our results suggest that the increased wettability of the Ti64-M-ASP/PLGA surface may contribute to better cell responses.

Figure 2E-a displayed that ASP revealed sustained release profile on the Ti64-M-ASP/PLGA surface during the 30-day observation period. The cumulative release amount reached 90% of the initial loaded ASP amount (Fig. 2E-b) until day 30. Moreover, daily concentration of ASP during the initial 25 days maintained within the range of 0.2–4 mM (Fig. 2E-c), which was proved to be the effective concentration to exert immunoregulatory function by our preliminary experiment (data not shown). The results reflected the controlled and stable released of ASP on the Ti64-M-ASP/PLGA surface. This may promote the conversion of macrophages from anti-inflammatory M1 type to pro-regenerative M2 type, and build the foundation for its immunoregulatory role to enhance osteoblast differentiation and osseointegration.

### Behaviors of RAW 264.7 cells cultured on different surfaces

#### Cell proliferation and cytotoxicity

**Fig. 3:**
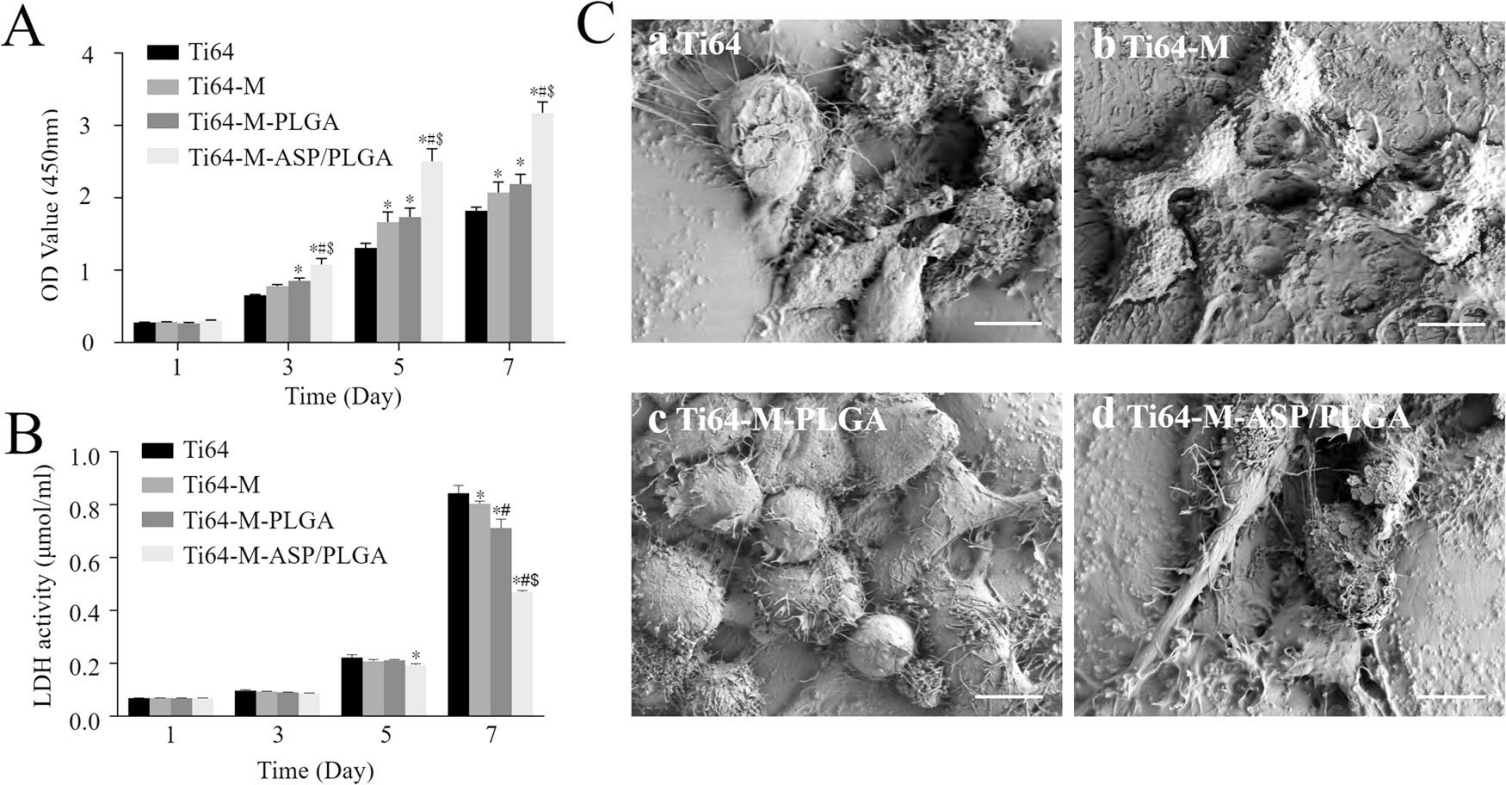
Effects of different samples on cell proliferation, cytotoxicity and morphology of RAW 264.7 macrophages. **A** CCK-8 and **B** LDH activity results of RAW 264.7 cells for 1, 3, 5 and 7 days. **C** SEM images of RAW 264.7 cells after 24 h of incubation. Data are expressed as the mean ± standard deviation (*n* = 3). ∗, #, and $ indicate statistical significance *p* < 0.05 *vs* Ti64, Ti64-M and Ti64-M-PLGA, respectively. Scale bars indicate 10 μm

The macrophages proliferation and viability on days 1, 3, 5 and 7 were evaluated by CCK-8 assay and LDH kit, respectively. As shown in Fig. 3A, cells displayed a progressive duplication for all the surfaces over the 7-day period. On day 5 and 7, all the other groups exhibited higher cell proliferation than the Ti64 group, with statistical differences (*p* < 0.05). Particularly, the Ti64-M-ASP/PLGA group evoked highest cell multiplication among all the surfaces, which was probably due to the duration of aspirin release, suggesting that addition of the ASP/PLGA coating can significantly promote cell proliferation. Apart from the action of aspirin, the concave structure of the surface of Ti64 provided a micrometer scale surface topography, which also played an important role in facilitating cell proliferation.

In addition, the LDH results (Fig. 3B) showed that cells survived on all the structures. There was no statistical difference from day 1 to 5 among groups, with only the Ti64-M-ASP/PLGA group displayed less cell apoptosis than the Ti64 group on day 5. On day 7, apoptotic cells in all groups increased significantly than those on day 5, which may be due to the rapid proliferation of macrophages to cause cell contact inhibition and induce apoptosis. It’s worth noting that the Ti64-M-ASP/PLGA group showed least apoptotic cell number, among all groups on day 7, implying the best cell survival, which might be correlated with the aspirin delivery of the ASP/PLGA coating. Based on our results, although all the surfaces possessed favorable cytocompatibility in vitro, the concave microstructure and ASP/PLGA coating, especially when combined, could promote cell proliferation and inhibit cell apoptosis to some extent.

#### Cell morphology

As shown in Fig. 3C, SEM images of RAW 264.7 cell on different surfaces were observed after 24 h of cultivation. Cells adhered to the Ti64 and Ti64-M surfaces looked spherical, with partially enlarged spreading areas, which indicate M1 polarization. While cells on the Ti64-M-PLGA substrate displayed well-spread shape, due to the property of PLGA to improve cell adhesion. Particularly, cells on the Ti64-M-ASP/PLGA substrate revealed elongated shape, which indicated a tendency towards the M2-polarized phenotype. It is well-accepted that M1 pro-inflammatory macrophage revealed round-shape with enlarged spreading area, while M2 pro-regenerative cells displayed slender shapes [41]. Therefore, our data revealed that Ti64-M-ASP/PLGA substrate could induce macrophages to transfer from anti-inflammatory M1 type to pro-regenerative M2 type. This phenomenon may be explained by that the sustained release of aspirin from the ASP/PLGA coating could exert immunoregulatory effects to shift the microenvironment around implant from inflammatory phase to regenerative phase.

#### Pro-inflammatory (M1) and pro-regenerative (M2) Marker Genes Expression

**Fig. 4:**
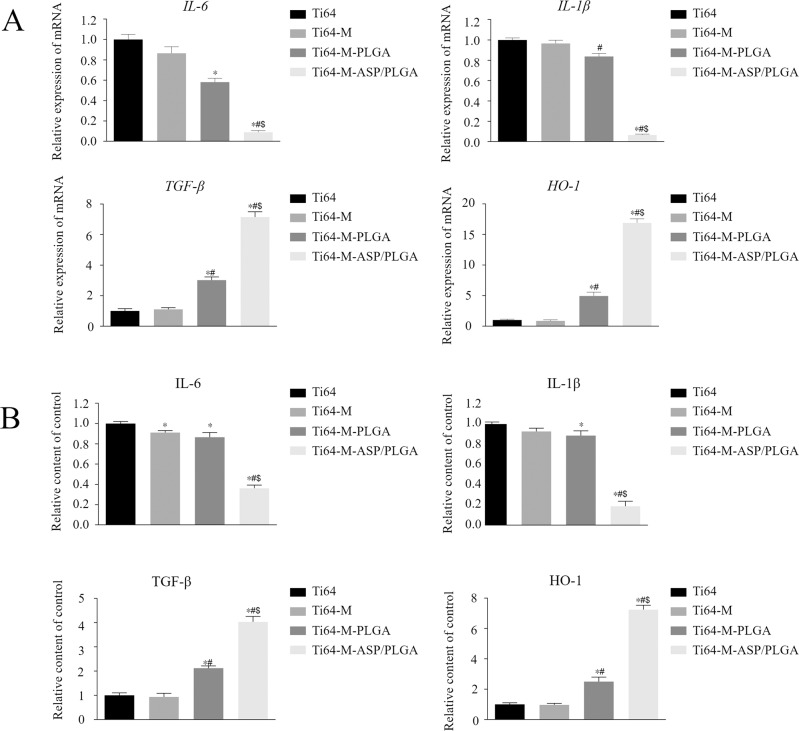
Relative pro-inflammatory (M1) and pro-regenerative (M2) marker genes and proteins expression in RAW 264.7 macrophages cultured with different samples. Gene expression levels (**A**) and Protein secretion level (**B**) of M1 and M2 markers after 3 days of incubation. Data are expressed as the mean ± standard deviation (*n* = 3). ∗, #, and $ indicate statistical significance *p* < 0.05 *vs* Ti64, Ti64-M and Ti64-M-PLGA, respectively

As shown in Fig. 4A, reduced M1 and increased M2 gene expressions were seen on the Ti64-M-PLGA surface, compared with the Ti64 group, *p* < 0.05. This suggested that the combined PLGA coating and micro-structured surface may inhibit inflammation and promote regeneration, to some extent. Most interestingly, the Ti64-M-ASP/PLGA substrate observed substantially elevated M2 and reduced M1 gene expressions, compared to all other groups, *p* < 0.05. When subjected to different stimuli, macrophages can switch between M1 and M2 phenotypes [42]. M1-type macrophages mainly secrete pro-inflammatory cytokine, such as IL-6, IL-1β, TNF-α, etc., while M2-type macrophages mainly secrete TGF-β, HO-1, IL-10 and other wound healing cytokines [19]. It was reported that after implant implantation, inflammation process lasts from several hours to several days [43], and the inflammatory cytokines start to decline 3 days post-implantation [44]. Therefore, we chose day 3 as the time point to detect gene expression change. These results proved that the ASP/PLGA coating could change macrophages from M1 to M2 phenotype, with obvious anti-inflammatory and pro-regenerative effects, so as to exert its excellent immunoregulatory function.

#### Enzyme-linked immunosorbent (ELISA) assay

We further checked the expression levels of pro-inflammatory (M1) and pro-regenerative (M2) marker proteins by ELISA assays (Fig. 4B). Similar with the gene expression tendency, for the Ti64-M-PLGA surface, the combination of micro-structure and PLGA evoked increased M2 proteins expression and slightly reduced M1 protein expression, compared to the Ti64 surface. Most importantly, on the Ti64-M-ASP/PLGA substrate, the M2 protein levels increased significantly, and the M1 protein levels obviously decreased. Our results implied that the Ti64-M-PLGA surface could exert the pro-regenerative and anti-inflammatory functions to some extent, while the Ti64-M-ASP/PLGA substrate could achieve pro-regenerative and anti-inflammatory effects remarkably, suggesting that the ASP/PLGA layer revealed its superior immunoregulatory function by transferring the LPS-induced macrophages from M1 inflammatory stage to M2 restorative stage.

### Behaviors of MC3T3-E1 cells on various surfaces in conditioned medium from macrophage

#### Cell proliferation and cytotoxicity

**Fig. 5:**
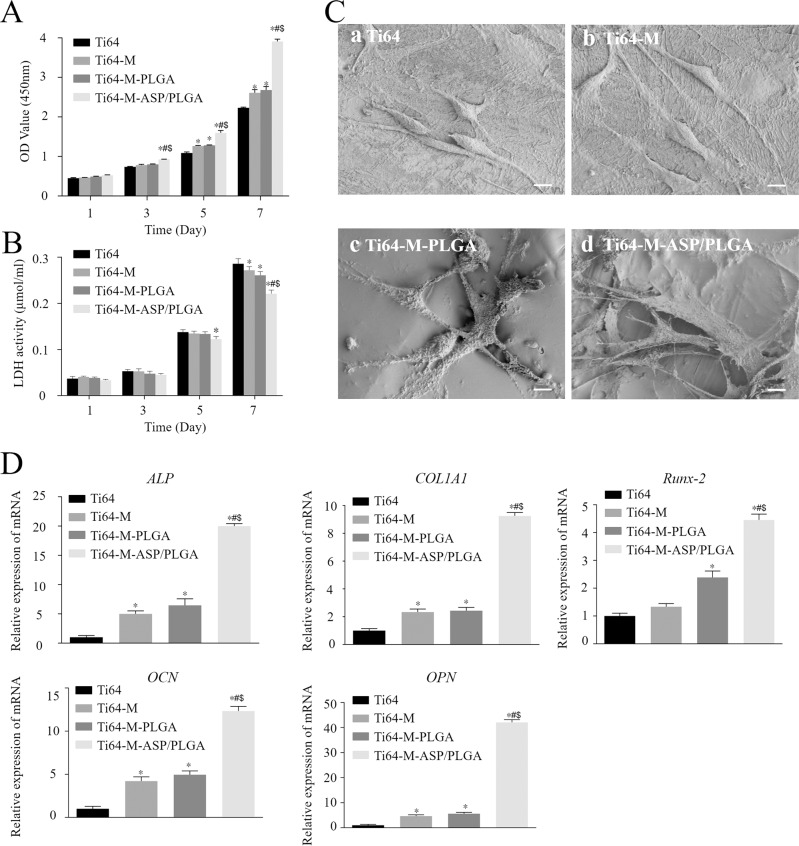
Effects of different samples on cell proliferation, cytotoxicity, morphology and osteogenesis-relate genes expression of MC3T3-E1 cells. **A** CCK-8 and **B** LDH activity results of MC3T3-E1 cells for 1, 3, 5 and 7 days. **C** SEM images of MC3T3-E1 cells after 24 h of culture. **D** Osteogenesis-related gene levels of MC3T3-E1 cells after 14 days of incubation. Data are expressed as the mean ± standard deviation (*n* = 3). ∗, #, and $ indicate statistical significance *p* < 0.05 *vs* Ti64, Ti64-M and Ti64-M-PLGA, respectively. Scale bar indicates 10 μm

Cell proliferation ability of MC3T3-E1 osteoblast cells seeded on different samples for 1, 3, 5 and 7 days was evaluated by CCK-8 kit. As shown in Fig. 5A, the cell number cultured on different surfaces revealed no statistically significance on day 1. On day 3, the cell proliferation on the Ti64-M-ASP/PLGA substrate was higher than the other groups, with statistical difference. While on day 5 and day 7, the Ti64-M and Ti64-M-PLGA group revealed higher cell proliferation than the Ti64 group. Particularly, the Ti64-M-ASP/PLGA substrate displayed the highest cell proliferation, with statistically significance.

The cytotoxicity of MC3T3-E1 cells seeded on different samples was evaluated by LDH kit and the results were shown in Fig. 5B. On day 1 and day 3, there was no statistical difference among different groups. On day 5, the Ti64-M-ASP/PLGA group showed lower cytotoxicity than the Ti64 group, with statistical difference. While on day 7, consistent with the cell proliferation results, the Ti64-M-ASP/PLGA substrate revealed the lowest cytotoxicity than the other surfaces, with statistically difference.

Our results showed that both PLGA and ASP could improve MC3T3-E1 cells proliferation and displayed no cytotoxicity. Most importantly, the Ti64-M-ASP/PLGA group showed best proliferative activity and least cytotoxicity among groups, indicating that aspirin released from the ASP/PLGA layer might improve osteogenesis at an early stage after implantation.

#### Cell morphology

After 24 h of incubation, the cell morphology of MC3T3-E1 cells on different samples were obtained by SEM as shown in Fig. 5C. Cells on Ti64 and Ti64-M surfaces both displayed spindle shape. While cells on the Ti64-M-PLGA surface revealed better spread-out morphology than the Ti64 and Ti64-M surfaces, with more filopodia and lamellipodia stretching out and adhered to the surfaces. Particularly, cells on the Ti64-M-ASP/PLGA substrate displayed the best well-spread morphology, with numerous well-distributed filopodia and lamellipodia extending in multiple directions and attached tightly into the underlying surface. It is reported that immune cells play vital roles in modulating bone dynamics, which can regulate the osteogenic microenvironment and promote osteogenesis [19]. Our results implied that the established Ti64-M-ASP/PLGA surface could promote osteoblast adhesion under the existence of macrophage CM, this might due to the anti-inflammatory effect of aspirin, which promotes osteogenic differentiation by regulating the transformation of macrophages to M2 type.

#### Osteogenesis-related genes expression

Figure 5D showed the expression of osteogenesis-related genes in osteoblasts after 14 days of incubation with various surfaces. The gene levels of *ALP*, *COL1A1*, *OCN* and *OPN* in cells cultured on the Ti64-M and Ti64-M-PLGA surfaces were higher than that on the Ti64 group (*p* < 0.05). This indicated that both micro structure and PLGA could promote osteogenesis to a certain degree. Remarkably, osteoblast cells on the Ti64-M-ASP/PLGA substrate revealed the highest levels of osteogenesis-related genes including *Runx-2*, *ALP*, *COL1A1*, *OPN* and *OCN* among all the surfaces, which implied the best osteogenic ability for the established Ti64-M-ASP/PLGA substrate. Previous study reported that the pro-regenerative M2 phenotype macrophages may promote osteogenesis in osteoblast cells [45]. Earlier in this article, we mentioned that Ti64-M-ASP/PLGA substrate invoked the shift from M1 towards M2 polarization in macrophages. Previous study also revealed that aspirin could inhibit macrophage activation in the early stages of inflammation and promote osteoblast differentiation and osteogenic activity [46]. Consistent with previous studies, the up-regulation of bone-related genes suggested that the ASP/PLGA coating layer of the Ti64-M-ASP/PLGA substrate could induce the shift to M2 phenotype macrophages, so as to improved osteogenesis in osteoblast cells.

#### Alizarin red S Staining

**Fig. 6:**
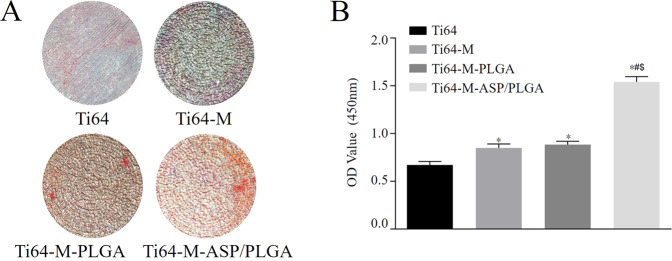
In vitro Alizarin red S staining in MC3T3-E1 cells cultured with different samples. **A** Alizarin red S staining and **B** Quantitative analysis after 21 days of culture. Data are expressed as the mean ± standard deviation (*n* = 3). ∗, #, and $ indicate statistical significance *p* < 0.05 *vs* Ti64, Ti64-M and Ti64-M-PLGA, respectively

Figure 6 showed the Alizarin red S staining photographs (Fig. 6A) and corresponding quantitative analysis (Fig. 6B) of MC3T3-E1 cells cultured on different surfaces for 21 days. It was revealed that both Ti64-M and Ti64-M-PLGA surfaces displayed more calcium nodules formation than the Ti64 group. In addition, the Ti64-M-ASP/PLGA substrate discovered most calcium nodules formation among all the groups. Mineralized calcium nodules are a sign of osteoblast differentiation and maturation, and it is also the main morphological manifestation of osteoblast to perform osteogenic function [47]. The largest amount of calcium nodules formation on the Ti64-M-ASP/PLGA substrate implied its highest osteogenic potential among all groups. The controlled release of aspirin from the ASP/PLGA coating contributed to the establishment of the pro-osteogenic niche. Our results further verified the superior osteogenic effects of the Ti64-M-ASP/PLGA substrate to induce osteoblast maturation and mineralization compared with other surfaces.

### Evaluation of bone formation in vivo

#### Push-out test and radiographic evaluation

**Fig. 7:**
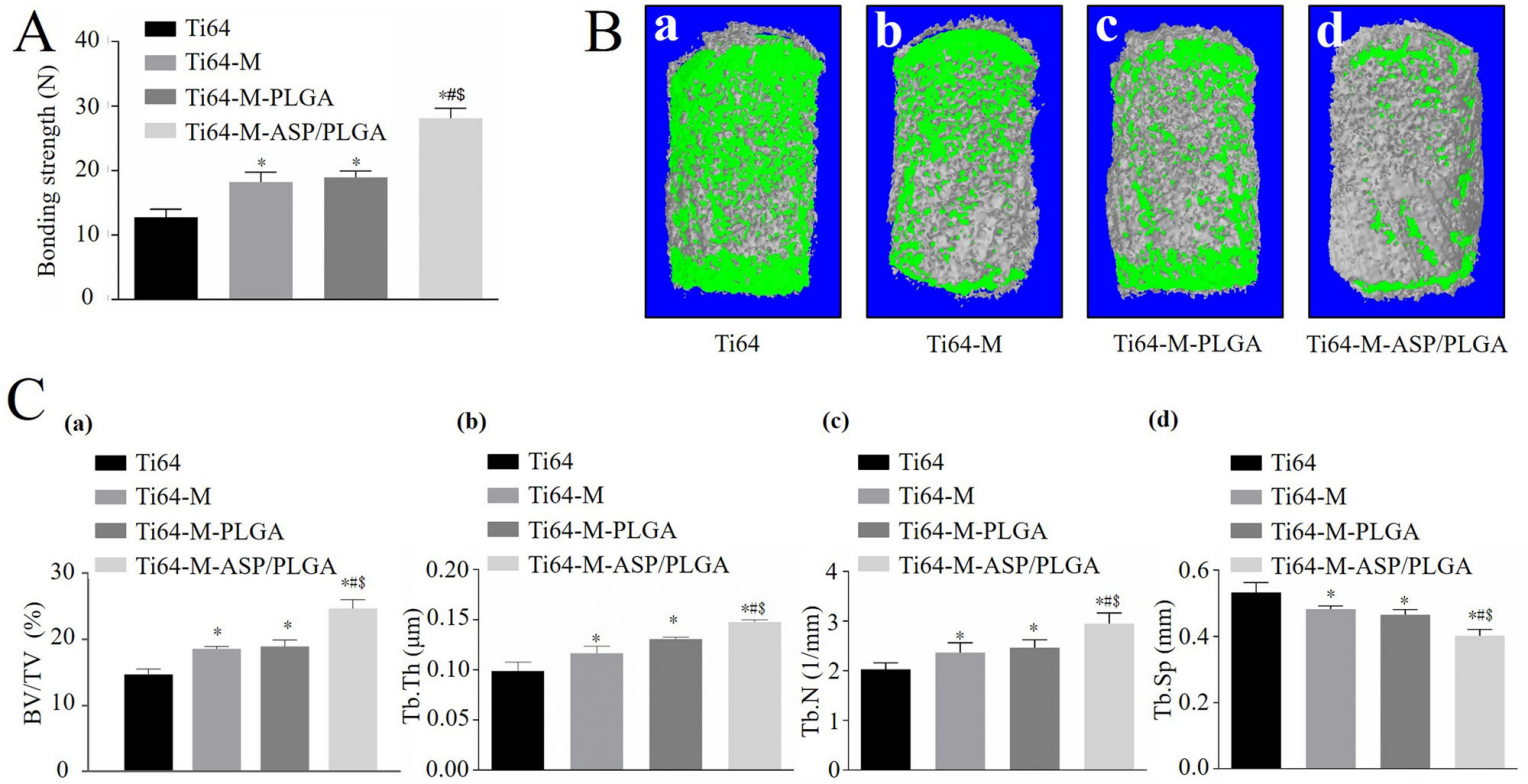
In vivo effects of implants with various surfaces in experimental rat model. After 4 weeks of implantation, biomechanical push out test (**A**), micro-CT 3D reconstruction (**B**) and quantitative analysis of BV/TV, Tb.Th, Tb.N and Tb.Sp (**C**) were performed. Data are expressed as the mean±standard deviation (*n* = 3). ∗, #, and $ indicate statistical significance *p* < 0.05 *vs* Ti64, Ti64-M and Ti64-M-PLGA, respectively

The bonding strength of bone-implant integration was evaluated by the biomechanical push-out test 4 weeks post-implantation. As shown in Fig. 7A, both Ti64-M and Ti64-M-PLGA groups detected higher push-out force values, compared to the Ti64 group, with statistical significance. Particularly, the Ti64-M-ASP/PLGA group showed the highest push-out force value among all groups. Our results suggested that both microstructure and PLGA could promote osseointegration, to some degree. Most importantly, superimposition of ASP on the micro-structured surface to construct the Ti64-M-ASP/PLGA substrate revealed the highest bone bonding ability in vivo. Thus, it was shown that the combination of ASP and PLGA can improve the bone binding strength of bone-implant interface to the highest extent.

Figure 7B demonstrated the reconstructed 3D images 4 weeks after insertion, with gray-colored new bone formed around the green-colored implants. The quantitative analyses were revealed in Fig. 7C. Among all groups, the highest BV/TV ratio and Tb.Th and Tb.N values were observed in the Ti64-M-ASP/PLGA group. While the lowest Tb.Sp value was seen for the Ti64-M-ASP/PLGA surface. Excellent bone-implant osseointegration is crucial for the fixation stability and long-term longevity [48]. Collectively, the Ti64-M-ASP/PLGA substrate observed the most favorable ability for bone ingrowth and maturation among all groups. Obviously, our results proved the satisfactory in vivo bone formation effects of the aspirin coating, which revealed the best osteogenic capability for the Ti64-M-ASP/PLGA group.

#### Histological assessments

**Fig. 8:**
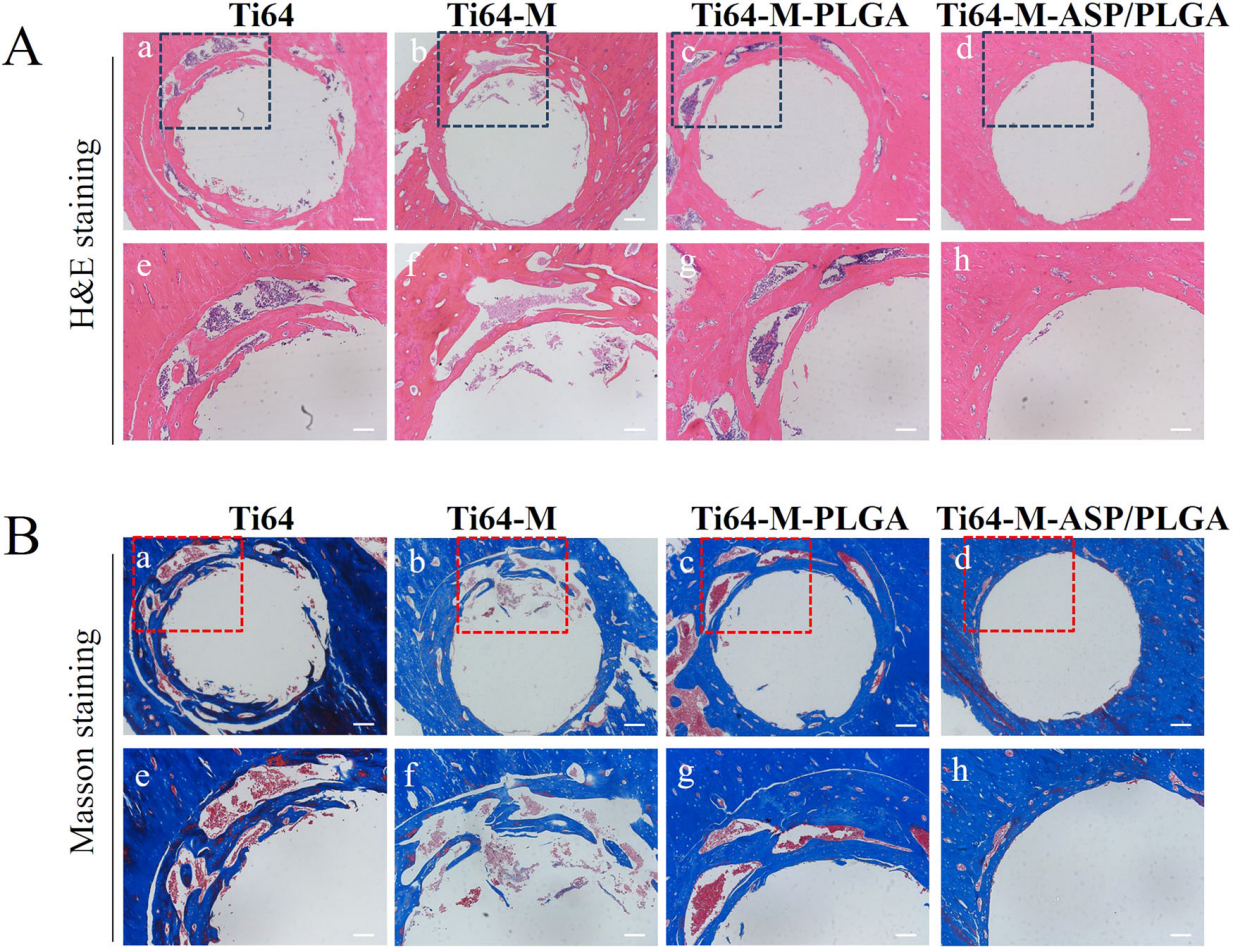
Bone formation ability evaluation using histological staining. **A** H&E and **B** Masson staining images after 4 weeks of implantation. Scale bars indicate 0.2 mm (a, b, c, d) and 0.1 mm (e, f, g, h) in both (**A**) and (**B**)

Figure 8 displayed the decalcified samples of peri-implant tissues around different groups stained with H&E and Masson staining to evaluate new bone formation. H&E staining in Fig. 8A showed that four weeks after implantation, relatively little amount of new osteoid tissue was seen around implants in the Ti64 group and Ti64-M group. While more new osteoid tissue was discovered in the Ti64-M-PLGA group than the Ti64 and Ti64-M groups. In particular, large amount of new osteoid tissue could be observed in the Ti64-M-ASP/PLGA group, indicating its superior bone formation ability. The Masson staining images (Fig. 8B) revealed similar tendency with the H&E staining results. Our results confirmed that the combination of concave microstructure and ASP/PLGA coating could synergistically promote bone formation, implying the best bone formation ability of the Ti64-M-ASP/PLGA substrate.

## Conclusion

In this study, we fabricated concave micro-structured 3D-printed Ti64 surface with ASP/PLGA composite coating to establish the Ti64-M-ASP/PLGA substrate. In vitro experiments demonstrated that concave micro-structure and ASP may exert synergistic effects to endow the established Ti64-M-ASP/PLGA substrate with good biocompatibility and the ability to subside inflammation and facilitate regeneration in macrophage cells. The timely shift from M1 to M2 phenotype macrophages enable the Ti64-M-ASP/PLGA substrate to display outstanding osteogenic ability in osteoblasts cultivated with CM from macrophage cells. More importantly, in vivo experiments indicated that the established Ti64-M-ASP/PLGA implants were in favor of bone ingrowth and maturation. In summary, this work offers a novel biocompatible implant surface, which superimposed the ASP/PLGA coating with the concave surface, to create ideal pro-osteogenic immune microenvironment for facilitating osteogenic ability in vitro and promoting osseointegration in vivo.

## References

[1] Krishna BV, Bose S, Bandyopadhyay A (2007). Low stiffness porous Ti structures for load-bearing implants [J]. Acta Biomaterialia.

[2] Hsu CS, Haag SL, Bernards MT, Li Q (2020). Evaluation of chlorine substituted hydroxyapatite (ClHAP)/polydopamine composite coatings on Ti64 [J]. Colloids Surf B, Biointerfaces.

[3] Bruinink A, Bitar M, Pleskova M, Wick P, Krug HF, Maniura-Weber K (2014). Addition of nanoscaled bioinspired surface features: A revolution for bone related implants and scaffolds? [J]. J Biomed Mater Res Part A.

[4] Yang BC, Zhou XD, Yu HY, Wu Y, Bao CY, Man Y (2019). [Advances in titanium dental implant surface modification] [J]. Hua xi kou qiang yi xue za zhi = Huaxi kouqiang yixue zazhi = West China J Stomatol.

[5] Kim HW, Koh YH, Li LH, Lee S, Kim HE (2004). Hydroxyapatite coating on titanium substrate with titania buffer layer processed by sol-gel method [J]. Biomaterials.

[6] Wang H, Su K, Su L, Liang P, Ji P, Wang C (2019). Comparison of 3D-printed porous tantalum and titanium scaffolds on osteointegration and osteogenesis [J]. Materials science & engineering C. Mater Biol Appl.

[7] Hayashi K, Munar ML, Ishikawa K (2020). Effects of macropore size in carbonate apatite honeycomb scaffolds on bone regeneration [J]. Materials science & engineering C. Mater Biol Appl.

[8] Wang H, Su K, Su L, Liang P, Ji P, Wang C (2018). The effect of 3D-printed Ti(6)Al(4)V scaffolds with various macropore structures on osteointegration and osteogenesis: A biomechanical evaluation [J]. J Mech Behav Biomed Mater.

[9] Zhang L, Yang G, Johnson BN, Jia X (2019). Three-dimensional (3D) printed scaffold and material selection for bone repair [J]. Acta Biomaterialia.

[10] Li Z, Liu C, Wang B, Wang C, Wang Z, Yang F (2018). Heat treatment effect on the mechanical properties, roughness and bone ingrowth capacity of 3D printing porous titanium alloy [J]. RSC Adv.

[11] Vrancken B, Thijs L, Kruth J-P, Van Humbeeck J (2012). Heat treatment of Ti6Al4V produced by Selective Laser Melting: Microstructure and mechanical properties [J]. J Alloy Compd.

[12] Mullen L, Stamp RC, Brooks WK, Jones E, Sutcliffe CJ (2009). Selective Laser Melting: a regular unit cell approach for the manufacture of porous, titanium, bone in-growth constructs, suitable for orthopedic applications [J]. J Biomed Mater Res Part B, Appl Biomater.

[13] Decuzzi P, Ferrari M (2010). Modulating cellular adhesion through nanotopography [J]. Biomaterials.

[14] Rabel K, Kohal RJ, Steinberg T, Tomakidi P, Rolauffs B, Adolfsson E (2020). Controlling osteoblast morphology and proliferation via surface micro-topographies of implant biomaterials [J]. Sci Rep.

[15] Wang X, Shah FA, Vazirisani F, Johansson A, Palmquist A, Omar O (2020). Exosomes influence the behavior of human mesenchymal stem cells on titanium surfaces [J]. Biomaterials.

[16] Chen Z, Klein T, Murray RZ, Crawford R, Chang J, Wu C (2016). Osteoimmunomodulation for the development of advanced bone biomaterials [J]. Mater Today.

[17] Wang J, Meng F, Song W, Jin J, Ma Q, Fei D (2018). Nanostructured titanium regulates osseointegration via influencing macrophage polarization in the osteogenic environment [J]. Int J Nanomed.

[18] Zhang R, Liu X, Xiong Z, Huang Q, Yang X, Yan H (2018). The immunomodulatory effects of Zn-incorporated micro/nanostructured coating in inducing osteogenesis [J]. Artif cells, Nanomed, Biotechnol.

[19] Chen Z, Bachhuka A, Wei F, Wang X, Liu G, Vasilev K (2017). Nanotopography-based strategy for the precise manipulation of osteoimmunomodulation in bone regeneration [J]. Nanoscale.

[20] He Y, Luo J, Zhang Y, Li Z, Chen F, Song W (2020). The unique regulation of implant surface nanostructure on macrophages M1 polarization [J]. Mater Sci Eng C, Mater Biol Appl.

[21] Minutti CM, Knipper JA, Allen JE, Zaiss DM (2017). Tissue-specific contribution of macrophages to wound healing [J]. Semin cell developmental Biol.

[22] Alkhalil M, Shahmohammadi M, Spence MS, Owens CG (2020). Aspirin Discontinuation in Patients Requiring Oral Anticoagulation Undergoing Percutaneous Coronary Intervention, The Role of Procedural Complexity [J]. Cardiovascular Drugs Ther.

[23] Wang Y, He G, Tang H, Shi Y, Kang X, Lyu J (2019). Aspirin inhibits inflammation and scar formation in the injury tendon healing through regulating JNK/STAT-3 signalling pathway [J]. Cell Prolif.

[24] Derry S, Moore RA (2012). Single dose oral aspirin for acute postoperative pain in adults [J]. Cochrane Database Syst Rev.

[25] Jiang Y, Wang SN, Wu HT, Qin HJ, Ren ML, Lin JC (2019). Aspirin alleviates orthopedic implant‑associated infection [J]. Int J Mol Med.

[26] Ren L, Pan S, Li H, Li Y, He L, Zhang S (2018). Effects of aspirin-loaded graphene oxide coating of a titanium surface on proliferation and osteogenic differentiation of MC3T3-E1 cells [J]. Sci Rep.

[27] Xu X, Gu Z, Chen X, Shi C, Liu C, Liu M (2019). An injectable and thermosensitive hydrogel: Promoting periodontal regeneration by controlled-release of aspirin and erythropoietin [J]. Acta Biomaterialia.

[28] Diouf A, Moufid M, Bouyahya D, Österlund L, El Bari N, Bouchikhi B (2020). An electrochemical sensor based on chitosan capped with gold nanoparticles combined with a voltammetric electronic tongue for quantitative aspirin detection in human physiological fluids and tablets [J]. Mater Sci Eng C, Mater Biol Appl.

[29] Ma AB, You YP, Chen B, Wang W, Liu J, Qi H (2020). Icariin/Aspirin Composite Coating on TiO2 Nanotubes Surface Induce Immunomodulatory Effect of Macrophage and Improve Osteoblast Activity [J]. Coatings.

[30] Yuan Z, Wei P, Huang Y, Zhang W, Chen F, Zhang X (2019). Injectable PLGA microspheres with tunable magnesium ion release for promoting bone regeneration [J]. Acta Biomaterialia.

[31] Danhier F, Ansorena E, Silva JM, Coco R, Le Breton A, Préat V (2012). PLGA-based nanoparticles: An overview of biomedical applications [J]. J Controlled Release.

[32] Yoshimoto I, Sasaki J-I, Tsuboi R, Yamaguchi S, Kitagawa H, Imazato S (2018). Development of layered PLGA membranes for periodontal tissue regeneration [J]. Dent Mater.

[33] Xia P, Wang S, Qi Z, Zhang W, Sun Y (2019). BMP-2-releasing gelatin microspheres/PLGA scaffolds for bone repairment of X-ray-radiated rabbit radius defects [J]. Artif cells, Nanomed, Biotechnol.

[34] Szewczenko J, Kajzer W, Kajzer A, Basiaga M, Kaczmarek M, Major R (2020). Influence of surface modification of Ti6Al7Nballoy on adhesion of poly (lactide-co-glycolide) coating (PLGA) [J]. Colloids Surfaces B-Biointerfaces.

[35] Keshavarz Shahbaz S, Foroughi F, Soltaninezhad E, Jamialahmadi T, Penson PE, Sahebkar A (2020). Application of PLGA nano/microparticle delivery systems for immunomodulation and prevention of allotransplant rejection [J]. Expert Opin Drug Deliv.

[36] Sun Y, Xing Z, Xue Y, Mustafa K, Finne-Wistrand A, Albertsson AC (2014). Surfactant as a critical factor when tuning the hydrophilicity in three-dimensional polyester-based scaffolds: impact of hydrophilicity on their mechanical properties and the cellular response of human osteoblast-like cells [J]. Biomacromolecules.

[37] Thomas M, Arora A, Katti DS (2014). Surface hydrophilicity of PLGA fibers governs in vitro mineralization and osteogenic differentiation [J]. Mater Sci Eng C, Mater Biol Appl.

[38] Hotchkiss KM, Clark NM, Olivares-Navarrete R (2018). Macrophage response to hydrophilic biomaterials regulates MSC recruitment and T-helper cell populations [J]. Biomaterials.

[39] Hotchkiss KM, Reddy GB, Hyzy SL, Schwartz Z, Boyan BD, Olivares-Navarrete R (2016). Titanium surface characteristics, including topography and wettability, alter macrophage activation [J]. Acta Biomaterialia.

[40] Zhou P, Mao F, He F, Han Y, Li H, Chen J (2019). Screening the optimal hierarchical micro/nano pattern design for the neck and body surface of titanium implants [J]. Colloids Surf B, Biointerfaces.

[41] Sadowska JM, Wei F, Guo J, Guillem-Marti J, Lin Z, Ginebra MP (2019). The effect of biomimetic calcium deficient hydroxyapatite and sintered β-tricalcium phosphate on osteoimmune reaction and osteogenesis [J]. Acta Biomaterialia.

[42] Mosser DM, Edwards JP (2008). Exploring the full spectrum of macrophage activation [J]. Nat Rev Immunol.

[43] Anderson JM, Mcnally AK (2011). Biocompatibility of implants: lymphocyte/macrophage interactions [J]. Semin Immunopathol.

[44] Zheng ZW, Chen YH, Wu DY, Wang JB, Lv MM, Wang XS (2018). Development of an Accurate and Proactive Immunomodulatory Strategy to Improve Bone Substitute Material-Mediated Osteogenesis and Angiogenesis [J]. Theranostics.

[45] Chen M, Huang L, Shen X, Li M, Luo Z, Cai K (2020). Construction of multilayered molecular reservoirs on a titanium alloy implant for combinational drug delivery to promote osseointegration in osteoporotic conditions [J]. Acta Biomaterialia.

[46] Liu Y, Fang S, Li X, Feng J, Du J, Guo L (2017). Aspirin inhibits LPS-induced macrophage activation via the NF-κB pathway [J]. Sci Rep.

[47] Zhu YS, Gu Y, Jiang C, Chen L (2020). Osteonectin regulates the extracellular matrix mineralization of osteoblasts through P38 signaling pathway [J]. J Cell Physiol.

[48] Brånemark R, Brånemark PI, Rydevik B, Myers RR (2001). Osseointegration in skeletal reconstruction and rehabilitation: a review [J]. J Rehabilitation Res Dev.

